# Increasing prevalence of thyroid autoimmunity in childhood type 1 diabetes in the pre-COVID but not during the COVID era

**DOI:** 10.3389/fendo.2024.1496155

**Published:** 2025-01-24

**Authors:** Vivien Herczeg, Eszter Muzslay, Diána Czipó, Lili Terkovics, Johanna Takács, Réka Garai, Fanni Kovács, Andrea Luczay, Anna Körner, Péter Tóth-Heyn

**Affiliations:** ^1^ Pediatric Center, Hungarian Academy of Sciences - Magyar Tudományos Akadémia (MTA) Center of Excellence, Semmelweis University, Budapest, Hungary; ^2^ Faculty of Medicine, Semmelweis University, Budapest, Hungary; ^3^ Department of Social Sciences, Faculty of Health Sciences, Semmelweis University, Budapest, Hungary; ^4^ Centre for Translational Medicine, Semmelweis University, Budapest, Hungary; ^5^ Diabetology Clinic, Szent János Hospital and North-Buda Unified Hospitals, Budapest, Hungary

**Keywords:** type 1 diabetes mellitus, COVID-19, epidemiology, Hashimoto disease, pediatric endocrinology, post-acute COVID-19 syndrome, SARS-CoV-2, autoimmune thyroiditis

## Abstract

**Introduction:**

Studies assessing longitudinal changes in the prevalence of autoimmune thyroiditis (AIT) among the pediatric population are limited. During the COVID-19 era, several papers proposed a rise in AIT cases. Our study aimed to analyze the prevalence of thyroid autoimmunity (TA) over a 10-year period spanning pre-pandemic and pandemic years in a population who are regularly screened for thyroid disturbances.

**Materials and methods:**

This single-center retrospective cohort study analyzed data from 1,361 children and young adults with type 1 diabetes (T1D) treated between 2013 and 2022 in Hungary’s largest pediatric endocrinology center. Results of anti-thyroid autoantibodies (anti-thyroid peroxidase/ATPO/and antithyroglobulin/ATG/), thyroid function tests (TFTs) and thyroid ultrasound examinations were obtained. Annual prevalence rates of TA and ultrasound-proven thyroiditis were calculated. Mean (± SD) follow-up period was 4.7 (± 2.8) years.

**Results:**

The overall prevalence of TA among our T1D children was 22.8% ([20.3;25.5], 310 cases) with significantly more girls affected (p<0.001). From 2013 to 2022, TA prevalence rose from 15.9% to 20.6% (p=0.041). The increase was detected during the pre-pandemic years but not in the COVID-19 era. Ultrasound-confirmed thyroiditis was present in 80.0% of examined TA cases. Ultrasound positivity rate was stable during the study period. Among our children with TA, 28.5% exhibited clinically relevant thyroid-stimulating hormone (TSH) abnormalities (most commonly subclinical hypothyroidism) and/or were prescribed thyroid medication. Children with AIT had a significantly elevated risk of thyroid dysfunction compared to those with only thyroid autoantibody positivity (p<0.001).

**Conclusion:**

Our results show a rise in the prevalence of thyroid autoimmunity among T1D children over the past decade, but our data do not support the assumed role of SARS-CoV-2 in the development of the disease.

## Introduction

During the coronavirus disease 2019 (COVID-19) pandemic era, numerous studies explored a potential connection between the Severe acute respiratory syndrome coronavirus 2 (SARS-CoV-2) and different autoimmune diseases. The majority of large clinical studies and meta-analyses reported an increase in the incidence or an acceleration in the progression of type 1 diabetes (T1D) during the pandemic and particularly, following SARS-CoV-2 infection (1–4). However, some studies presented opposing results or found that the rise during the pandemic was within the range of the expected incidence (5–7). Similarly, while several studies indicated a rise in thyroid autoimmunity (TA, presence of anti-thyroid autoantibodies) and autoimmune thyroiditis (AIT, positive autoantibodies with inflammation of the thyroid gland as proven by ultrasound) during the pandemic, evidence remains controversial (8–12). Two studies specifically investigated the prevalence of TA in newly diagnosed T1D children, however, they also reported contradictory findings (13, 14). Our research group has also examined the prevalence of thyroid disturbances among the pediatric population post-COVID-19 but we - like many other scientists - faced a major limitation: the scarcity of pre-pandemic control data (15).

Type 1 diabetes (T1D) is the most common childhood endocrine disease. It is well-established that individuals with T1D have an elevated risk of developing other autoimmune conditions, such as AIT. According to the literature, the prevalence of AIT in children with T1D ranges from 17 to 30%, highlighting the strong association between these two autoimmune disorders (16).

As pediatric patients with T1D are at higher risk of developing AIT [most commonly Hashimoto’s disease (17)], they undergo regular screening for TA based on the recommendation of the International Society for Pediatric and Adolescent Diabetes’ [ISPAD, most recently updated in 2022 (18)]. To better understand the impact of the COVID-19 pandemic, we aimed to collect and analyze longitudinal data on AIT and TA in our pediatric T1D population.

The primary purpose of our study was to determine the annual prevalence of TA and ultrasound-proven thyroiditis among pediatric patients with T1D treated in one of Central Europe’s largest pediatric endocrinology-diabetology centers over a 10-year period, covering both pre-pandemic (2013-2020) and pandemic (2021-2022) years. Additionally, we aimed to assess longitudinal trends in anti-thyroid peroxidase (ATPO) and antithyroglobulin (ATG) antibody titers along with thyroid function test (TFTs) results and data on thyroid medication during this period.

## Materials and methods

### Study setting, data collection and extraction

Our single-center retrospective cohort study was conducted at the Bókay Unit of the Pediatric Center, Semmelweis University, Budapest, Hungary. This center is responsible for the care of approximately 25% of all T1D children and adolescents in the country. Before exclusion, we obtained all patients’ data with diabetes mellitus (DM) who received insulin treatment and had at least one visit between 1^st^ of January, 2013 and 31^st^ of December, 2022 at the inpatient and/or outpatient Endocrinology and Diabetes Unit of our Center. Clinical data and laboratory results were obtained from Semmelweis University’s e-MedSolution software. Medical records were retrieved based on the BNO classification system, the Hungarian adaptation of the ICD-10 (The International Statistical Classification of Diseases and Related Health Problems, 10^th^ Revision) classification released by the World Health Organization (https://icd.who.int/browse10/2019/en, accessed: 09.10.2024).

Data was completed and verified by four medical doctors and two medical students in agreement with our pre-specified research protocol. All arising questions were discussed in detail within the data extraction team.

Our study was conducted in accordance with the World Medical Association’s Declaration of Helsinki. Due to the retrospective nature of our study, ethical approval was not required.

### Inclusion and exclusion criteria

Inclusion criteria: all patients between 0 to 21 years of age (hereafter referred to collectively as “children”), who had been treated with T1D at our center between 2013 and 2022, and had at least one simultaneous ATPO and ATG measurements during our study period. For the purposes of this study, we have chosen 21 years as the upper age limit, consistently with our clinical practice, where care is provided to individuals up to 21 years of age. Data on TA were obtained from both newly diagnosed and follow-up cases.

Exclusion criteria: patients over 21 years of age, children with diabetes forms other than T1D, and those who never underwent TA screening throughout the duration of our study.

### Measured parameters and assay methodology

We collected the following parameters: clinical data of children (sex, date of birth, T1D diagnosis date), visit dates, thyroid autoantibody (ATPO, ATG) and TFTs (thyroid-stimulating hormone/TSH/, free thyroxine/fT4/) results from all years when the patient was screened as well as thyroid ultrasound findings for antibody-positive patients. Mean ages and time since T1D diagnosis were calculated according to the mid-point of each calendar year (1^st^ of July). Laboratory analysis were carried out at the Immunology Laboratory of Semmelweis University with assay methodology and normal ranges shown in **Table 1**. If a child had more than one laboratory examination of a parameter in a calendar year, we used the last data for our analysis (or the last one before they started taking thyroid medicines in case of TSH and fT4). TSH levels above 6 mU/L or below 0.1 mU/L, and/or results that were consistently abnormal in a patient, were considered clinically relevant. TA was defined by at least one positive ATPO and/or ATG laboratory result obtained during our study period. Assay methodology and ranges of normal values of autoantibodies had changed multiple times over the examined period, therefore we calculated both absolute and relative values. Thyroid ultrasounds were deemed positive for thyroiditis if they showed enlargement and/or inhomogeneity and/or hyperemia in the thyroid gland. AIT was diagnosed if at least one autoantibody was positive and thyroiditis was confirmed by ultrasound examination.

**Table 1:** Laboratory assay methodology and ranges of normal values.

Laboratory parameter	Test used during the study period	Assay methodology, manufacturer	Units of measurement	Lower limit	Upper limit
Anti-thyroid peroxidase (ATPO)	1st of January, 2013 - 24th of March, 2014	ELISA, Aesku	UI/mL	–	40
	25th of March, 2014 - 20th of January, 2017	ECLIA, Roche	U/mL	–	63
	21st of January, 2017 - 11th of January, 2022	CLIA, Abbott	U/mL	–	5.6
	12th of January, 2022 - 31st of December, 2022	ECLIA, Roche	U/mL	9	34
Antithyroglobulin (ATG)	1st of January, 2013 - 24th of March, 2014	ELISA, Aesku	UI/mL	–	120
	25th of March, 2014 - 31st of December, 2022	ECLIA, Roche	UI/mL	–	115
Thyroid-stimulating hormone (TSH)	1st of January, 2013 - 31st of December, 2022	CLIA, Siemens	mU/L	0.35	4.94
Free thyroxine (fT4)	1st of January, 2013 - 31st of December, 2022	CLIA, Siemens	pmol/L	9	23.2

CLIA, Chemiluminescence Immunoassay; ECLIA, Electro-chemiluminescence Immunoassay; ELISA, Enzyme-linked Immunosorbent Assay.

### Assessment of autoantibody results

Screening frequency of children was primarily based on ISPAD’s guidelines (18), therefore patients without thyroid comorbidity did not go through autoantibody measurements each year. However, if a patient had presented a positive thyroid autoantibody result, they had been examined in all years afterwards. Due to the different screening frequencies between positive and negative children, prevalence data would be unrealistically high if not adjusted. Thus, in years when no laboratory examinations were carried out, we used the following protocol:

If the antibody titers were negative in both the preceding and subsequent years’, we considered the intermediate year to be negative as well. When both were positive, we recognized it as positive. If they differed, the intermediate year was administered as missing data.

### Description of the pre-pandemic and pandemic periods

We defined the pre-pandemic period as 2013 to 2020 and the pandemic period as 2021 and 2022. In the year of 2020, we did not expect a significant impact of the pandemic on our children, as a large Hungarian national representative study indicated that there were very few pediatric COVID-19 cases during the first pandemic wave (19).

### Statistical analysis

Statistical analysis was carried out by a professional biostatistician. Each year, we calculated the prevalence using data from all children under care at that time. Consequently, the data for most children are included in our database for multiple years, corresponding to each year they were under care at our center. To calculate and compare prevalence data, and perform descriptive statistics, MedCalc Statistical Software version 22.023 was used (MedCalc Software bv, Ostend, Belgium; https://www.medcalc.org; 2020). Chi square comparison were conducted using StataCorp. (Stata Statistical Software: Release 18.5). The visualization was created using Microsoft Excel (Microsoft Corporation, 2016).

## Results

### Demographics

Between January 1, 2013 and December 31, 2022, a total of 1,667 patients with insulin-dependent DM were treated at our Clinic. We excluded 14 adults over 21 years old, 65 children who had another condition rather than T1D and 227 patients due to missing data and/or who were treated primarily at other centers. All in all, we included 1,361 T1D children’s data in our final analysis. Mean (± SD) follow-up period was 4.7 (± 2.8) years. The girl-to-boy ratio was 637:724 (46.8% *vs* 53.2%). Detailed annual data are provided in **Table 2**.

**Table 2:** Characteristics of included children.

	2013	2014	2015	2016	2017	2018	2019	2020	2021	2022
Number of patients	483	513	520	631	680	732	689	738	745	713
Girls: boys (%)	48.2: 51.8	46.8: 53.2	48.5: 51.5	47.7: 52.3	46.9: 53.1	47.4: 52.6	45.9: 54.1	47.2: 52.8	45.9: 54.1	47.7: 52.3
Mean (SD) age (years)	12.1 (4.1)	12.3 (4.1)	12.5 (3.9)	12.3 (4.1)	12.4 (4.1)	12.4 (4.2)	12.4 (4.1)	12.4 (4.2)	12.5 (4.3)	12.6 (4.3)
Mean (SD) time from T1D diagnosis (years)	5.0 (3.8)	5.1 (3.9)	5.3 (3.9)	5.1 (3.9.)	5.2 (3.9)	5.3 (4.0)	5.3 (3.9)	5.4 (3.9)	5.6 (4.1)	5.7 (4.0)

T1D, type 1 diabetes.

### Thyroid autoimmunity and ultrasound-confirmed thyroiditis

The overall prevalence of thyroid autoimmunity among our T1D children was found to be 22.8% ([20.3;25.5], 310 children). Girls were significantly more affected. (TA group: 106 boys, 204 girls, non-TA group: 618 boys, 433 girls; p<0.001, RR: 1.60 [1.43;1.78]).

**Figure 1:**
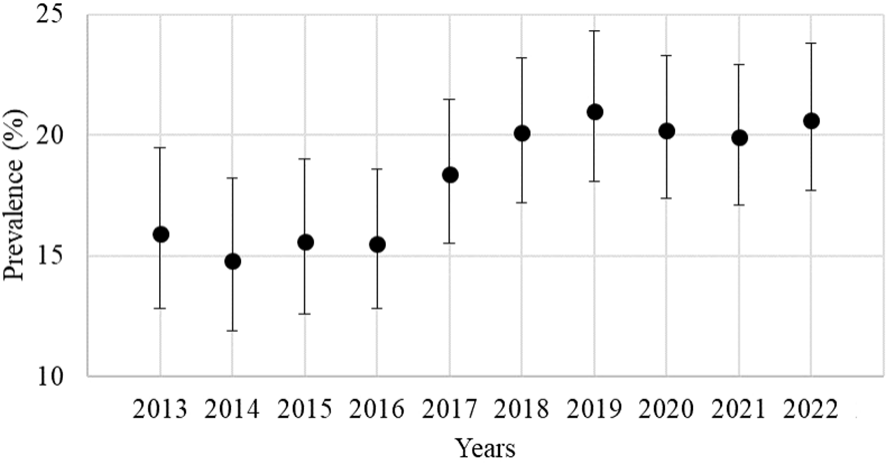
Annual prevalence rate of thyroid autoimmunity.

From 2013 to 2022, the prevalence of TA increased from 15.9% to 20.6% (p=0.041). We observed this increase during the pre-pandemic years (2013 to 2019) but not in the COVID-19 era. Whilst analyzing the yearly prevalence differences, we found no significant difference between any consecutive years. Annual prevalence rates with confidence intervals (CI) are shown in **Figure 1**.

Of the children with TA who were screened with ultrasound at least once (n=260), 208 (80.0%) showed signs of thyroiditis on ultrasound, representing 67.1% of all children with TA. This resulted in an overall AIT prevalence of 15.3% in our study group. Among the 208 children with ultrasound-proven thyroiditis, one was later diagnosed with papillary thyroid carcinoma, as was one other child who did not show signs of thyroiditis. Although we performed an increasing number of ultrasounds throughout the years, the positivity rate remained stable. Yearly ATPO, ATG and ultrasound positivity rates can be seen in **Table 3**.

**Table 3:** Thyroid autoimmunity results.

	2013	2014	2015	2016	2017	2018	2019	2020	2021	2022
Prevalence of TA (%, ratio)	15.9 (77/483)	14.8 (76/513)	15.6 (81/520)	15.5 (98/631)	18.4 (125/680)	20.1 (147/732)	21.0 (145/689)	20.2 (149/738)	19.9 (148/744)	20.6 (147/713)
US positivity rate of children with TA (%, ratio)	81.8 (9/11)	82.8 (24/29)	85.4 (35/41)	82.0 (50/61)	88.8 (71/80)	79.8 (83/104)	75.2 (85/113)	79.4 (85/107)	83.1 (98/118)	83.5 (96/115)
Positivity rate of ATG (%, ratio)	3.8 (9/236)	9.8 (28/285)	14.0 (41/292)	12.0 (59/490)	12.0 (75/627)	12.6 (81/642)	11.5 (33/287)	6.7 (31/463)	11.3 (50/441)	9.6 (47/490)
Positivity rate of ATPO (%, ratio)	14.4 (35/243)	15.1 (46/304)	15.3 (52/340)	11.2 (58/518)	17.3 (102/591)	18.3 (118/644)	16.8 (66/392)	15.0 (76/505)	16.7 (74/443)	13.8 (68/493)

TA, thyroid autoimmunity; US, ultrasound; ATG, antithyroglobulin; ATPO, anti-thyroid peroxidase.

Given the multiple changes in assay methodology of ATPO and ATG titers during our examined period, we calculated both absolute and relative values in case of both antibodies. Detailed results of autoantibody titers are provided in **Supplementary Tables 1**, **2**.

### Clinically relevant thyroid dysfunction

We also analyzed the rate of thyroid hormone changes and data related to thyroid medication over this 10-year period in our children with TA. From the TFTs’ analysis, we excluded children who did not have at least one TSH result during the study period (n=8). For children who were prescribed thyroid medication, we included only data preceding the initiation of treatment. Therefore, children who started their medication before 2013 (the beginning of our study period) were also excluded (n=20). Among the included 282 children, 44 (15.6%) exhibited clinically relevant elevation in TSH levels, while eight (2.8%) showed TSH reduction during the study period. Increased fT4 levels were observed in four cases, while decreased fT4 levels were noted in two children. The annual results are presented in **Table 4**. Detailed description of TSH and fT4 values can be seen in **Supplementary Table 3**.

**Table 4:** Annual results of thyroid function tests among children with thyroid autoimmunity.

		2013	2014	2015	2016	2017	2018	2019	2020	2021	2022
TSH results (%, number)	Low	0.0 (0)	0.0 (0)	3.3 (3)	1.6 (2)	0.0 (0)	0.6 (1)	0.0 (0)	0.8 (1)	0.8 (1)	0.8 (1)
	Normal	79.5 (31)	92.7 (38)	95.6 (86)	96.1 (122)	98.7 (152)	95.9 (162)	98.6 (138)	96.2 (126)	91.3 (116)	94.4 (119)
	High	20.5 (8)	7.3 (3)	1.1 (1)	2.4 (3)	1.3 (2)	3.6 (6)	1.4 (2)	3.1 (4)	7.9 (10)	4.8 (6)
	All	39	41	90	127	154	169	140	131	127	126
fT4 results (%, number)	Low	2.6 (1)	0.0 (0)	0.0 (0)	0.8 (1)	0.0 (0)	0.0 (0)	0.7 (1)	0.0 (0)	0.8 (1)	0.0 (0)
	Normal	97.4 (38)	100.0 (40)	100.0 (90)	99.2 (121)	100.0 (153)	100.0 (169)	99.3 (139)	99.2 (130)	99.2 (125)	99.2 (125)
	High	0.0 (0)	0.0 (0)	0.0 (0)	0.0 (0)	0.0 (0)	0.0 (0)	0.0 (0)	0.8 (1)	0.0 (0)	0.8 (1)
	All	39	40	90	122	153	169	140	131	126	126

TSH, thyroid-stimulating hormone; fT4, free thyroxine.

Among the 310 children with TA, 74 (23.9%) were treated for hypo- and/or hyperthyroidism. Of these, 63 were initially prescribed levothyroxine to manage subclinical or clinical hypothyroidism, while two required levothyroxine following a total thyroidectomy due to thyroid carcinoma. Additionally, nine children were originally treated with antithyroid (thyrostatic) medications (thiamazole or propylthiouracil). During our study period, three children required treatment with both types of medication. Two of them began with thiamazole and later transitioned to levothyroxine due to hypothyroidism, while one child started on levothyroxine and subsequently began taking thiamazole.

Altogether, out of the 302 children with TA who had at least one TSH result during the study period, 86 (28.5%) had clinically relevant TSH abnormalities and/or were prescribed thyroid medication.

### The association between ultrasound findings and thyroid function results

We examined the connection between ultrasound positivity and thyroid function abnormalities among the children with at least one ultrasound and one TSH result (n=258). From this analysis, we excluded the two aforementioned children with papillary thyroid carcinoma, as they had started hormone replacement therapy following total thyroidectomy. The contingency table summarizing these data is presented as **Table 5**. Children with ultrasound-proven thyroiditis had a significantly elevated risk of having TSH abnormalities and/or being prescribed thyroid medication (p<0.001, RR: 6.24 [2.05;18.98]).

**Table 5:** Association between ultrasound positivity (thyroiditis) and thyroid function abnormalities.

	TSH Abnormalities/Medication	No TSH Abnormalities/Medication	Total number
Positive ultrasound result	76	131	207
Negative ultrasound result	3	48	51
Total number	79	179	258

TSH, thyroid-stimulating hormone.

## Discussion

In our retrospective cohort study involving 1,361 children and young adults with T1D, we found an increase in the prevalence of TA from 15.9% to 20.6% between 2013 and 2022. Interestingly, this increase was notable in the pre-COVID period (2013-2020) but not during the pandemic years (2021-2022). While the study’s primary aim was to explore the potential effect of the COVID-19 pandemic on the prevalence of TA among T1D children, our results revealed a previously underreported trend: the rise in TA prior to the pandemic.

Numerous data shows that the prevalence rates of autoimmune diseases are increasing (20–22). A population-based cohort study of 22 million individuals in the UK found the largest incidence rate increase in coeliac disease, Sjogren’s syndrome and Graves’ disease, while Hashimoto’s thyroiditis significantly decreased in incidence from 2000 to 2019 (23).

Studies involving the frequency of TA in pediatric T1D populations, mostly report on pooled prevalence data (17, 24). Cumulative incidence rate of TA was reported in children with T1D in a recent French study covering a similar study period like ours (2014 to 2020). They found an overall thyroid autoantibody frequency of 18% (25). The cumulative incidence of TA at 10 years of T1D was found to be 14% in a former study (26). However, longitudinal data on the trend of TA prevalence in the pediatric T1D population is scarce.

High prevalence of autoimmune thyroid diseases (AITDs) has been linked to various genetic, epigenetic, and environmental factors, though evidence on their specific effects remains inconsistent. Altered DNA methylation patterns, which regulate gene expression without altering the DNA sequence, may play a role in AITD development (27). Some studies have identified a relationship between vitamin D deficiency or insufficiency and AITDs (28, 29), while others found no such connection (30, 31). Additionally, insufficient selenium intake has been implicated in Hashimoto’s disease occurrence (32). Higher iodine consumption has also been associated with an increased presence of anti-thyroid antibodies (33, 34). Stress-related disorders significantly elevated the risk of subsequent AITD, with a hazard ratio of 1.49 (1.42–1.56) observed in a large Swedish cohort study (35). However, the prospective Amsterdam AITD Cohort study found no link between stress exposure and *de novo* appearance of ATPO (36). Furthermore, an increasing number of studies are investigating the role of endocrine-disrupting chemicals (EDCs) in the onset and severity of AITDs, though research in this area remains limited (37). Among infectious agents, besides SARS-CoV-2, Epstein–Barr virus, Parvovirus B19, Human Herpesvirus 6A and Helicobacter pylori have all been suggested as contributors to AITD development (38–41).

Our hypothesis was that due to the high pediatric SARS-CoV-2 infection rate, we would observe an even higher TA prevalence among T1D children during the pandemic period. Our result do not support this initial hypothesis and it should be added, that even if we would have found an increasing prevalence during the pandemic period, our study design would only allow us to describe an association but not to state a cause-effect relationship. With the analysis of the pre-pandemic and pandemic era, we only had the opportunity to investigate the hypothesized change of the cumulative effect of numerous possible influencing factors (impact of SARS-CoV-2 and COVID-19 vaccines, delays in diagnosis making, changes in numerous habits such as eating, taking supplements, physical activity, decrease of other viral infections due to the restrictive measures and also other aspects which are not related to the pandemic and were discussed earlier).

To the best of our knowledge, only a few studies compared TA prevalence among T1D children between pre-pandemic and pandemic era with only one reporting annual data as well (14). Our results are in line with a small Turkish study, which found no significant difference in the frequency of concurrent thyroid autoantibodies (neither in ATPO nor in ATG) in newly diagnosed T1D children during the first pandemic year (February 2020 - January 2021) in comparison to the previous three years (February 2017 - January 2020) (13). In contrast, Al-Abdulrazzaq et al. published opposing results from Kuwait: children diagnosed with T1D during the COVID-19 pandemic (February 2020 - December 2022) had double the odds of testing positive for thyroid antibodies compared to those diagnosed earlier (January 2017 - February 2020). Additionally, according to their results, children with a positive history of COVID-19 were more likely to present with thyroid antibodies. In contrast to our finding of an increasing trend during the pre-pandemic years, they documented a stable prevalence between 2017 and 2019 (14).

Several studies reported an increased rate of TA during and after COVID-19 in adult, non-T1D populations. Increased prevalence of latent TA (ATPO positivity) was described in patients hospitalized for COVID-19 compared to healthy, pre-pandemic controls (8). Rossini et al. described a doubled prevalence of autoimmune thyroid disease (increased ATPO) in COVID-19 survivor adults compared to age and sex-matched controls (15.7% vs 7.7%) (9). An observational study from Spain documented a doubled diagnosis rate of Graves’ disease in 2021, compared to data from 2017-2020 (10). In contrast, Lui et al. found only incidental TA positivity among adults recovering from COVID-19 (11). Moreover, a retrospective cohort study of pregnant women showed no significant difference in the incidence of new-onset thyroid dysfunction or autoimmunity between those who were SARS-CoV-2 seropositive and those who were seronegative postpartum (12). In a recent comprehensive review on the topic, the authors concluded that although SARS-CoV-2 can affect the thyroid gland and possibly cause subacute thyroiditis or TA, according to follow-up studies, survivors of COVID-19 showed no substantial long-term thyroid sequelae (42).

This issue is much less discussed in the pediatric literature than in adult studies. According to a recent narrative review focusing on the impact of the COVID-19 pandemic on thyroid diseases in the general pediatric population, there is currently no conclusive evidence linking SARS-CoV-2 with an increased incidence of TA in children. Additionally, they highlighted the paucity of data on the youth (43). A retrospective analysis by Shidid et al. observed no significant differences in the percentage of abnormal TSH results between the pre-pandemic (January 2017–October 2019) and pandemic periods (March 2020–October 2021). They focused on thyroid dysfunction without reporting on the frequency of TA (44). Consistently, a retrospective observational study from a tertiary pediatric endocrine center in the United Kingdom found no substantial changes in the presentation of thyroid dysfunction (hypo- and hyperthyroidism) in children before and after the pandemic. McCowan et al. also highlighted that the COVID-19 pandemic had no significant effect on fT4 or ATPO in hypothyroid patients (45). It is evident that there is a need for more pediatric-specific research.

Recent studies suggest that COVID-19-related thyroid dysfunction may occur through multiple mechanisms. SARS-CoV-2 can potentially disrupt thyroid function either by directly damaging thyroid cells via angiotensin-converting enzyme 2 (ACE-2) receptors or through indirect effects, such as triggering or exacerbating autoimmune processes (46–48). The virus may trigger autoimmune responses through various mechanisms, including molecular mimicry of SARS-CoV-2 proteins, bystander activation, epitope spreading during tissue damage, disruption of immunoprivileged barriers, polyclonal lymphocyte activation, and pathological activation of antigen-presenting cells via superantigens (48).

Our results support the findings of numerous previous large studies reporting female predominance among T1D children with TA (14, 17, 21, 24, 26). Additionally, older age and longer diabetes duration might increase the risk of developing TA (14, 24). In our study, the mean of these parameters remained stable during our study period so these factors did not bias our results.

Though the ISPAD protocol does not require ultrasound examinations for diagnosis and monitoring TA, we are proud that the number of performed ultrasound examinations grew remarkably during our study period and we could cover the vast majority of children among our TA population by 2022. It should be noted, that the positivity rate of ultrasound examinations was stable throughout the whole study period. We find this diagnostic tool inevitable in the early detection of malignancies.

Furthermore, we aimed to examine the differences between AIT and TA. While AIT specifically refers to an autoimmune condition in which the thyroid gland shows evidence of inflammation, TA is a broader term that encompasses the presence of thyroid-specific autoantibodies without necessarily having inflammation detectable by ultrasound. We found that children with inflammation of the thyroid gland (AIT) had a significantly greater likelihood of thyroid function abnormalities and subsequent thyroid medication use compared to those with only thyroid-specific autoantibodies and negative ultrasound results (TA). However, it is noteworthy that three children exhibited TSH alterations despite having no signs of thyroiditis on their ultrasound examinations.

### Strengths

The main strength of our study is that it covers ten years of annual data on more than 1,300 children allowing us to assess the prevalence of TA longitudinally. The decade-long study period enables for a comprehensive assessment of trends in TA over time. By analyzing TA prevalence on a year-by-year basis, the study provides a more nuanced understanding of the trends compared to pooled data analysis. It has the ability to reflect on the increasing trend during a long-term period, while with the comparison of pre-pandemic and pandemic groups, one might assume that the difference between the pooled prevalences could be attributed to the impact of the pandemic. The inclusion of both pre-pandemic and pandemic years adds an important dimension to the understanding of the trends before and during the COVID-19 pandemic. Additionally, given that our center is responsible for the care of about 25% of all T1D children in Hungary and is one of the largest in Central Europe, we believe that our findings are representative of this region.

### Limitations

As this was a retrospective study, we encountered instances of incomplete or missing data in certain cases. Possible reasons for these were missed annual check-ups by some patients, variations in protocols among specialists, overlooked screenings or insufficient blood sample volumes. Furthermore, the documentation regarding medication may have been inconsistent. Moreover, the laboratory test method used for the measurement of ATPO and ATG changed multiple times during our study period, thus we needed to present relative values of ATPO and ATG besides absolute ones. Unfortunately, as to our experiences, neither of them is ideal for precise comparison, nevertheless we indicated both results. Finally, it should be noted, that autoimmune diseases may develop months or years after an infection, so our study (ending in 2022) might not capture the long-term effects of the pandemic.

### Conclusion

In our retrospective cohort study, we found an increase in the prevalence of thyroid autoimmunity among children with type 1 diabetes from 2013 to 2022. Unexpectedly, this growth was remarkable during the studied pre-pandemic period and it just stopped during the COVID-19 pandemic years. Therefore, our results among T1D children do not support the assumed role of SARS-CoV-2 in the development of thyroid autoimmunity.

## Data Availability

The raw data supporting the conclusions of this article will be made available by the authors, without undue reservation.
