# Supplementary material for: Increasing prevalence of thyroid autoimmunity in childhood type 1 diabetes in the pre-COVID but not during the COVID era
Source: Front Endocrinol (Lausanne). 2025 Jan 24;15:1496155. doi: 10.3389/fendo.2024.1496155 (PMC11803430; doi:10.3389/fendo.2024.1496155)
Supplement: Supplementary file 1 [file Table1.pdf]

Supplementary Table 1. Descriptive statistics of autoantibody titers of all T1D children

	**2013**				**2014**			
	Absolute ATPO	Relative ATPO	Absolute ATG	Relative ATG	Absolute ATPO	Relative ATPO	Absolute ATG	Relative ATG
Mean	64.70	1.57	33.84	0.28	89.55	1.71	84.82	0.74
SD	203.00	5.00	208.45	1.74	275.21	5.67	376.29	3.27
Median	6.00	0.15	2.00	0.02	9.21	0.15	12.10	0.11
25th percentile	2.00	0.05	1.00	0.01	5.30	0.09	10.00	0.09
75th percentile	11.00	0.28	4.00	0.03	21.08	0.36	18.65	0.16

	**2015**				**2016**			
	Absolute ATPO	Relative ATPO	Absolute ATG	Relative ATG	Absolute ATPO	Relative ATPO	Absolute ATG	Relative ATG
Mean	68.27	1.08	90.87	0.79	61.16	0.97	76.26	0.66
SD	192.22	3.05	330.62	2.87	276.86	4.39	268.97	2.34
Median	8.92	0.14	14.05	0.12	10.85	0.17	14.25	0.12
25th percentile	5.90	0.09	10.58	0.09	7.57	0.12	10.00	0.09
75th percentile	16.97	0.27	22.20	0.19	17.96	0.29	23.03	0.20

	**2017**				**2018**			
	Absolute ATPO	Relative ATPO	Absolute ATG	Relative ATG	Absolute ATPO	Relative ATPO	Absolute ATG	Relative ATG
Mean	79.97	12.62	110.43	0.96	88.64	15.83	113.26	0.98
SD	335.46	52.92	931.00	8.10	401.41	71.68	815.35	7.09
Median	0.21	0.04	10.60	0.09	0.16	0.03	10.00	0.09
25th percentile	0.16	0.03	10.00	0.09	0.16	0.03	10.00	0.09
75th percentile	1.31	0.18	17.00	0.15	1.25	0.22	15.28	0.13

	2019				2020			
	Absolute ATPO	Relative ATPO	Absolute ATG	Relative ATG	Absolute ATPO	Relative ATPO	Absolute ATG	Relative ATG
Mean	84.45	15.08	75.84	0.66	88.46	15.80	52.24	0.45
SD	355.15	63.42	301.91	2.63	561.07	100.19	253.34	2.20
Median	0.46	0.08	10.00	0.09	0.44	0.08	13.00	0.11
25th percentile	0.16	0.03	10.00	0.09	0.16	0.03	11.10	0.10
75th percentile	1.43	0.26	13.98	0.12	1.29	0.23	15.80	0.14

	2021				2022			
	Absolute ATPO	Relative ATPO	Absolute ATG	Relative ATG	Absolute ATPO	Relative ATPO	Absolute ATG	Relative ATG
Mean	114.97	19.79	63.11	0.55	55.38	1.62	60.90	0.53
SD	695.95	123.23	193.57	1.68	238.84	7.01	231.74	2.01
Median	0.65	0.12	16.10	0.14	9.00	0.26	13.60	0.12
25th percentile	0.16	0.03	13.60	0.12	9.00	0.26	12.40	0.11
75th percentile	2.01	0.36	20.25	0.18	14.05	0.41	15.50	0.13

ATG: antithyroglobulin, ATPO: anti-thyroid peroxidase

Supplementary Table 2. Descriptive statistics of autoantibody titers of T1D children with positive thyroid autoantibodies

	2013				2014			
	Absolute ATPO	Relative ATPO	Absolute ATG	Relative ATG	Absolute ATPO	Relative ATPO	Absolute ATG	Relative ATG
Mean	406.65	9.84	769.22	6.41	484.08	9.38	699.25	6.08
SD	383.73	9.62	800.85	6.67	521.03	11.14	994.32	8.65
Median	260.00	6.50	593.00	4.94	249.95	4.08	287.40	2.50
25th percentile	146.40	2.97	410.00	3.42	140.73	2.28	210.80	1.83
75th percentile	604.00	14.11	741.00	6.18	625.83	11.10	649.00	5.64

	2015				2016			
	Absolute ATPO	Relative ATPO	Absolute ATG	Relative ATG	Absolute ATPO	Relative ATPO	Absolute ATG	Relative ATG
Mean	351.08	5.57	542.37	4.72	443.41	7.04	504.44	4.39
SD	350.95	5.57	742.18	6.45	724.08	11.49	627.67	5.46
Median	257.90	4.09	278.20	2.42	263.50	4.18	316.20	2.75
25th percentile	131.10	2.08	175.10	1.52	149.75	2.38	216.95	1.89
75th percentile	392.60	6.23	544.60	4.74	421.60	6.69	460.40	4.00

	2017				2018			
	Absolute ATPO	Relative ATPO	Absolute ATG	Relative ATG	Absolute ATPO	Relative ATPO	Absolute ATG	Relative ATG
Mean	458.78	72.74	810.74	7.05	481.86	86.05	792.54	6.89
SD	694.36	109.31	2601.21	22.62	833.42	148.83	2188.67	19.03
Median	194.88	34.08	285.30	2.48	196.88	35.16	327.00	2.84
25th percentile	54.21	9.10	194.15	1.69	53.89	9.62	204.60	1.78
75th percentile	602.91	85.51	505.80	4.40	561.15	100.20	557.50	4.85

	2019				2020			
	Absolute ATPO	Relative ATPO	Absolute ATG	Relative ATG	Absolute ATPO	Relative ATPO	Absolute ATG	Relative ATG
Mean	497.12	88.77	544.02	4.73	583.13	104.13	555.55	4.83
SD	740.77	132.28	744.55	6.47	1348.81	240.86	839.82	7.30
Median	168.51	30.09	304.10	2.64	91.53	16.34	290.10	2.52
25th percentile	44.10	7.87	229.00	1.99	19.44	3.47	212.65	1.85
75th percentile	731.11	130.55	465.60	4.05	445.44	79.54	475.20	4.13

	2021				2022			
	Absolute ATPO	Relative ATPO	Absolute ATG	Relative ATG	Absolute ATPO	Relative ATPO	Absolute ATG	Relative ATG
Mean	678.14	116.70	406.21	3.53	330.64	9.72	470.81	4.09
SD	1587.25	282.25	443.93	3.86	569.47	16.75	610.88	5.31
Median	173.18	30.17	238.40	2.07	120.50	3.54	273.00	2.37
25th percentile	42.42	7.58	184.48	1.60	71.08	2.09	184.00	1.60
75th percentile	544.86	85.82	427.45	3.72	376.25	11.07	524.50	4.56

ATG: antithyroglobulin, ATPO: anti-thyroid peroxidase

Supplementary Table 3. Thyroid function tests' results among children with thyroid autoimmunity

		2013	2014	2015	2016	2017	2018	2019	2020	2021	2022
Thyroid stimulating hormone (TSH)	Mean	3.93	2.66	2.39	2.35	2.32	2.38	2.34	2.82	3.36	2.91
	SD	3.41	1.80	1.62	1.55	1.49	2.28	1.31	1.46	3.38	2.62
	Median	2.42	2.26	1.95	2.07	2.09	1.92	2.03	2.50	2.53	2.51
	25th percentile	1.74	1.44	1.43	1.40	1.34	1.29	1.48	1.80	1.83	1.55
	75th percentile	4.51	3.40	3.27	2.98	2.76	2.76	2.89	3.41	3.81	3.63
Free thyroxine (fT4)	Mean	13.26	13.08	12.96	13.00	13.09	13.00	12.74	12.65	12.94	13.99
	SD	2.00	1.42	1.90	1.68	1.75	1.65	1.57	2.08	1.63	2.30
	Median	13.02	13.18	12.91	13.00	13.07	12.97	12.67	12.52	12.74	13.61
	25th percentile	12.01	12.10	11.47	11.70	12.01	11.75	11.69	11.45	11.90	12.59
	75th percentile	14.39	14.09	13.86	14.01	14.11	14.03	13.75	13.46	14.12	14.95
